# Wedelolactone-Loaded Micelles Ameliorate Doxorubicin-Induced Oxidative Injury in Podocytes by Improving Permeability and Bioavailability

**DOI:** 10.3389/fbioe.2019.00333

**Published:** 2019-11-22

**Authors:** Liang Feng, Zhi-yong Li, Long Wang, Xing-hua Li, Ya-ping Chen, Bing Yang, Dang Yang, Yuan-pei Lian, Xue-feng Hou, Jun-hui Li, Shu-min Ding, Xiao-bin Jia

**Affiliations:** ^1^School of Traditional Chinese Pharmacy, China Pharmaceutical University, Nanjing, China; ^2^China Minority Traditional Medical Center, Minzu University of China, Beijing, China; ^3^Affiliated Hospital of Integrated Traditional Chinese and Western Medicine, Nanjing University of Chinese Medicine, Nanjing, China; ^4^Department of Nephrology, Shanghai JiaoTong University Affiliated Sixth People's Hospital, Shanghai, China; ^5^School of Pharmaceutical Engineering & Life Science, Changzhou University, Changzhou, China

**Keywords:** wedelolactone, micelles, nephrotoxicity, oxidative stress, podocytes

## Abstract

Wedelolactone (WED) is commonly used for the treatment of doxorubicin (DOX)-induced kidney damage, but its efficacy is limited by its poor solubility and bioavailability. In this study, we developed a novel delivery system of WED-loaded micelles (WED-M) with Solutol^®^ HS15 and lecithin at an optimized ratio of 7:3 to improve the poor permeability and bioavailability of WED and to enhance its efficacy. The spherically shaped WED-M (particle size: 160.5 ± 3.4 nm; zeta potential: −30.1 ± 0.9 mV; entrapment efficiency: 94.41 ± 1.64%; drug loading: 8.58 ± 0.25%; solubility: 1.89 ± 0.06 mg/ml) has continuous stability over 14 days and a sustained release profile. The permeability of WED-M in Caco-2 cells indicated a significant 1.61-fold higher Papp AP to BL ratio than WED alone. Additionally, pharmacokinetic evaluation of WED-M demonstrated that the bioavailability of WED was increased 2.78-fold. Both HE staining and transmission electron microscopy showed an obvious improvement of pathological damage in WED-M treatment. Moreover, WED-M significantly enhanced the ROS level in mice and MPC5 podocytes. We concluded that using this micelle delivery system for WED could improve its permeability and bioavailability to attenuate DOX-induced oxidative injury in podocytes. This study provided important information on the fact that the micelle delivery system, WED-M, showed a significant improvement of renal damage.

## Introduction

Oxidative stress is a common mechanism of kidney injury. The widely used Doxorubicin (DOX, a potent chemotherapy agent) has been regarded as a crucial contributor in the pathogenesis of oxidative stress in nephrotoxicity injury. The Quinolone cycle caused by DOX can produce excessive reactive oxygens (ROS), such as superoxide, hydrogen peroxide, and hydroxyl radical, and therefore induce the oxidative stress (Ramesh and Mandal, 2019; Vasconcelos et al., 2019). Wedelolactone (WED) is an ingredient in Eclipta alba (CCE) that could significantly alleviate nephrotoxicity injury induced/produced by oxidative stress (Ghosh et al., 2017; AboulFotouh et al., 2019) and has a potential to protect against DOX-induced nephrotoxicity (Zhu et al., 2019).

However, the use of WED to protect the kidney from injury caused by oxidative stress has been limited due to its poor solubility and low biological availability. The permeability and bioavailability of the drug affect its efficacy on mitochondrial (ROS)-mediated injury. Therefore, the Wedelolactone-loaded micelles (WED-M) drug delivery system was developed to improve the unique physicochemical properties of permeability and bioavailability and was evaluated on the basis of its pharmacokinetics, pharmacodynamics, and biological distribution (Nair et al., 2019). There is potential evidence that the prepared WED micelles can enhance oral bioavailability.

Lecithin is a common natural material that is often used in liposome-based delivery (Peng et al., 2018). It has a lot of advantages for the construction of nano-carriers. Polymeric micelles have recently been applied for the delivery of hydrophobic drugs. In particular, Solutol^®^ HS15, a surfactant that self-assembles in micelles developed by BASF SE, has been very popular among many researchers due to its low toxicity and strong solubilization effect. Furthermore, mixed micelles, self-assembled from two or more dissimilar block copolymers, have usually shown a better solubilization effect than the single micelles in many reports (Li and Tan, 2008). Therefore, we elected to combine lecithin with Solutol^®^ HS15 to produce a mixed micelle with high drug solubility and low toxicity.

The purpose of this study is: (1) to present the preparation method for WED-M micelle; (2) to evaluate whether Solutol^®^ HS15 and lecithin increase WED permeability and bioavailability; (3) to evaluate the improvement effect of WED-M and WED on oxidative stress injury; (4) to provide an innovative approach to improve bioavailability.

## Materials and Methods

### Reagents and Chemicals

Wedelolactone was provided by Chengdu Pufei De Biotech Co., Ltd. (purity > 98.0%, Chengdu, China). Prednisone acetate was from Huazhong Pharmaceutical Co., Ltd. (Xiangyang, China). Doxorubicin hydrochloride for injection was obtained from KeyGen. Co. Ltd. (Nanjing, China). Lecithin (average molecular weight: 760 g/mol) was purchased from Shanghai Advanced Vehicle Technology Pharmaceutical Ltd (Shanghai, China). Solutol^®^ HS15 (molecular weight: 963 g/mol) was purchased from Sigma-Aldrich (St. Louis, MO, USA). Acetic acid, acetonitrile, and methanol were Chromatographic Grade and were obtained from Tedia Company Inc. (Fairfield, USA). A 2′,7′-dichlorofluorescein diacetate (DCFDA)-cellular ROS detection assay kit was obtained from Abcam (Cambridge, UK). The other reagents are analytical.

### Preparation of WED-Loaded Mixed Micelles

**Figure 1:**
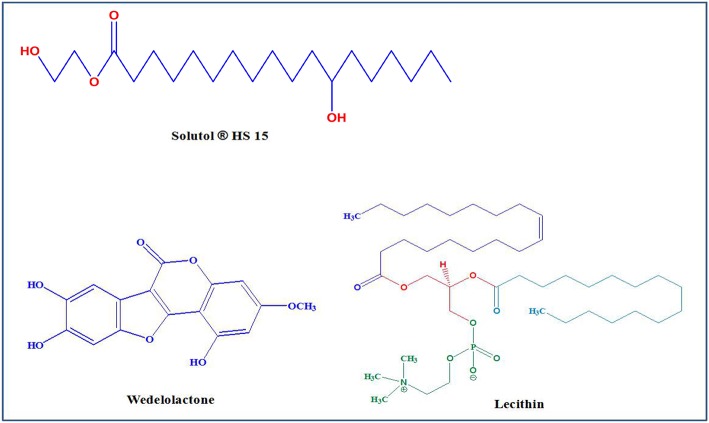
Chemical structure of Solutol^®^ HS 15, Wedelolactone (WED), and Lecithin.

WED-loaded binary mixed micelles were prepared by a thin-film hydration method (Zhu et al., 2017). In brief, 60 mg of WED and a mixture of Solutol^®^ HS15 and lecithin (600 mg) at a ratio of 7:3 (w/w) were dissolved in absolute ethyl alcohol by the ultrasonic method in a round-bottom flask (Figure 1). The ethanol in the solution was removed at 45°C under a vacuum of 0.1 MP. The product was then rehydrated in deionized water of 30 ml to obtain a clear solution. Next, the solution was filtered through a 0.45 μm membrane filter to remove the non-incorporated WED. The mixed micelles of WED-M were stored at 4°C for further experiments. The mixed micelles prepared by this method can be well-dispersed in water.

### Morphology, Zeta Potential, and Particle Size of WED-Loaded Mixed Micelles

The zeta potential and particle size of the WED-M were measured by dynamic light scattering with a Malvern system (ZEN-3600, Malvern Instruments, Worcestershire, UK). The particle size distribution of the particles was evaluated by the polydispersity index (PDI). Examination of the morphology of the WED-M was performed by transmission electron microscopy (TEM, JEM-2100, JEOL, Japan) after negative staining with sodium phosphotungstate solution (2%, w/v).

### WED Loading Efficiency and Encapsulation Ratio

Determination of the concentration of WED in the mixed micelles was performed by the high-performance liquid chromatography method (Hou et al., 2017). In short, 200 μl of WED-M was dissolved and the phospholipid was disrupted by adding 800 μl methanol. The mixture was centrifuged at 22,380 × g for 10 min and filtered through a 0.45 μm membrane filter before being transferred into the HPLC. Compared with the concentration of WED in the micelle (>1 mg/mL), the concentration of WED in deionized water (<1 μg/mL) is negligible because of its poor solubility. Thus, free WED in the micelle solution can be ignored when calculating the drug loading (DL%) and encapsulation efficiency (EE%).

The equations of calculating EE% (1) and DL% (2) are as follows.

(1) $$\begin{array}{lll} W_{\text{EE}} & = & m_{1}/m_{2}\times 100\% \end{array}$$

(2) $$\begin{array}{lll} W_{\text{DL}} & = & m_{1}/\left(m_{2}+m_{3}\right)\times 100\% \end{array}$$

m_1_: the quality of the drug in the micellar solution; m_2_: the quality of the drug added; m_3_: the quality of the carrier material.

### Storage Stability

After being stored at 4°C for 1, 3, 7, and 14 days, the drug solubility, average particle size, clarity, zeta potential, and PDI of the micelle systems were investigated for evaluating the storage stability of WED-M.

### *In vitro* Release Study

The *in vitro* release behavior of WED-M was studied using the dialysis method. Namely, WED-M and WED suspensions (170 μg/mL, 1 mL) were divided into experimental groups and a control group. Each 1 mL WED-M or WED suspension was placed in a dialysis bag (molecular weight cutoff 3,500 g/mol; Green Bird Inc., Shanghai, China). The dialysis bags were immersed in fresh 50 mL PBS (pH 7.4, 37°C) and 0.5% (w/v) Tween 80 (Sinopharm Chemical Reagent Co., Ltd.). The solubility of WED-M is 1.89 ± 0.06 mg/mL, so the release medium with a volume of 50 mL conforms to the sink condition. This medium was stirred at 100 rpm for 48 h. At predetermined time points (0.5, 1, 2, 4, 8, 12, 24, 36, and 48 h), an equal volume of fresh release medium was used to replace 1 mL of the release media to maintain a constant volume. The content of WED released from the release medium was detected by the HPLC method.

### Caco-2 Cell Culture

Caco-2 cells were purchased from KeyGen Co. Ltd. (Nanjing, China) and were used for the WED transport experiment. Caco-2 cells were seeded on Transwell R-6 well-permeable supports (25,000 cells/ml) in an incubator at 37°C with a humidified atmosphere (95% relative humidity) containing 5% CO_2_. The cells were well-developed, with TEER values >350 Ω × cm^2^ 21 days post-seeding.

### Transport Experiment

Before transport assays, differentiated cell monolayers were washed with HBSS (pH 7.4, 37°C) three times and incubated at 37°C for 30 min. Sequentially, a solution-dissolved WED-M or WED (20 μmol/L) and blank HBSS was added to the apical (AP) or basolateral (BL) side, respectively. Donor samples and receiver samples (400 μL) were taken at 0, 1, 2, 3, and 4 h time points for detection. The transport experiment was stopped by 100 μL of methanol containing 5 μM testosterone (internal standard, IS) and then centrifuged at 22,380 × g for 5 min at 4°C. The supernatant containing WED was measured by the HPLC method. All experiments were performed in triplicate. The TEER value was tested to ensure the survival of Caco-2 cell monolayers. The penetrability of WED was calculated with equation (El-Sayed et al., 2017):

$$\begin{array}{l} {\text{P}}_{\text{app}}\text{AP}\to \text{BL}=\left(\text{dC}/\text{dt}\right)\left(1/\text{SC}\right)\\ {\text{P}}_{\text{app}}\text{BL}\to \text{AP}=\left(\text{dM}/\text{dt}\right)\left(1/\text{SC}\right) \end{array}$$

where V represents the volume of the receiver (2.5 mL), S represents the surface area of the cell monolayer (4.2 cm^2^), C represents the original concentration, dC/dt represents the rate of concentration change in the side of receiver, and dM/dt represents the rate of drug transport. Linear regression analysis was used to obtain the rate of WED transport.

### Pharmacokinetic Comparison

In the study of pharmacokinetics, SD rats were randomly separated into two groups (*n* = 6) and orally administered WED and WED-M at a dose of 50 mg/kg. After administration, blood samples were collected from the orbital vein at predetermined time points (0, 0.25, 0.5, 0.75, 1, 2, 3, 4, 5, 6, 8, 12, and 24 h). After taking blood, the rats were euthanized by carbon dioxide asphyxia. The blood samples were centrifuged immediately to obtain the plasma. Four hundred and fifty microliter of methanol was added to the plasma and vigorously vortexed for 5 min to precipitate the protein. The samples were then centrifuged at 20,660 ×g for 10 min at 4°C. After centrifugation, 20 μL of the supernatant was injected into UPLC-MS/MS to determine the concentration of WED.

### Animal Model and Treatment Protocols

Male ICR mice (weight, 20 ± 2 g) were provided by SLAC Laboratory Animal Co., Ltd. (certificate NO. SCXK 2015-0002). All animals were allowed to acclimate for 1 week at a temperature of 24 ± 1°C and humidity of 60% ± 5%. All mice were housed in cages with food and tap water *ad libitum*. All experiments were performed in accordance with relevant guidelines and regulations approved by the Experimental Animal Research Committee of China Pharmaceutical University. The protocol was approved by the institutional animal ethics committees of China Pharmaceutical University.

Mice were randomly divided into six groups (six animals/each): control, doxorubicin (DOX) alone, DOX plus prednisone acetate (PSL, 10 mg/kg, i.g), DOX plus WED (25, 50 mg/kg, i.g), DOX plus WED-M (25, 50 mg/kg, i.g). A 7.5 mg/kg dosage of DOX was given once intravenously to mice to induce kidney injury, as previously described (Zhu et al., 2014). Mice in the control group were injected with normal saline instead. Seven days (on day 8) after DOX injection, mice were administered with WED, WED-M and prednisone acetate and kept for 28 days. Animals in the control and DOX only groups were administered with the same volume of normal saline.

### MPC5 Cell Culture and Treatment

Conditionally immortalized murine podocytes (MPC5) were provided by KeyGEN Biological Technology Development Co., Ltd. (Nanjing, China). Cells were seeded in a plastic culture flask containing DMEM with 10% FBS, 4 mM of L-glutamine, 100 U/ml of penicillin, and 100 U/ml of streptomycin and then placed in a humidified incubator containing 5% CO_2_ at 37°C. The medium was replaced every 2 days. After 90% confluence had been achieved, the cells were digested using a 0.25% trypsin-0.02% EDTA solution. Cells with a density of 1 × 10^6^/ml were prepared for the experiment.

*In vitro*, podocyte injury was performed in MPC5 cells according to previous reports (Feng et al., 2013). Cells were treated with basal DMEM medium for the blank control group. DOX-treated cells were divided into five groups (*n* = 6/group): a model group (4 μM DOX), positive control group (4 μM DOX plus 10 μM dexamethasone), low dose of WED-M group (4 μM DOX plus 1.25 μM WED-M), medium dose of WED-M group (4 μM DOX plus 5 μM WED-M), and high dose of WED-M group (4 μM DOX plus 20 μM WED-M). Cells were cultured in an incubator at 37°C with 5% CO_2_ for 24 h.

### Biochemical Analysis

On days 3, 7, 14, 21, 28, and 35 after DOX injection, mice in each group were put into metabolic cages, and 24-h urine samples were collected for measurement of urinary protein. On day 36, the blood of the mice was taken orbital intravenously and was centrifuged at 5,165 ×g for 10 min. After taking the blood, the mice were euthanized by cervical vertebra dislocation. The supernatant of the blood was drawn carefully to obtain the serum. The serum was isolated, and the levels of serum albumin (ALB), serum total protein (TP), total cholesterol (TC), triglyceride (TG), serum creatinine (Scr), and blood urea nitrogen (BUN) were measured by automatic biochemical analyzer. Twenty-four hours of urinary protein excretion was measured by urine protein test kits according to the manufacturer's protocols.

### Histological Study

Kidney tissues were carefully stripped and rinsed in normal saline for subsequent experiments. Kidney tissue was fixed in 10% formalin, embedded in paraffin and stained with hematoxylin and eosin (HE), and assessed under light microscopy. Sections were examined by a qualified and blinded pathologist to evaluate the degree of pathological changes based on glomerular epithelial hyperplasia, tubular dilatation, and protein cast formation. These sections were scored from 0 to 10 (normal to severe). The total score for each renal sample was calculated. The remaining kidney was used for electron microscopic examination. Small blocks of renal tissues were fixed in 2.5% glutaraldehyde, postfixed in 2% osmium tetroxide, dehydrated in graded ethanol and acetone, and then embedded in epoxy resin. Ultrathin sections were stained with lead citrate and uranyl acetate and examined using an electron microscope (JEM-1400, Japan). The renal cortex was stored at −80°C.

### Determination of Reactive Oxygen Species (ROS)

The level of ROS was detected using a 2′,7′-dichlorofluorescein diacetate-cellular ROS detection assay kit according to our previous method (Li et al., 2013). Briefly, the cells were treated with drug molecules and loaded with 10 μM DCFDA under darkness at 37°C for 30 min and then washed with PBS three times. The fluorescence was imaged by fluorescence spectroscopy (Olympus IX71, Japan) with excitation of 495 nm and emission of 525 nm.

### Flow Cytometry

Intracellular ROS generation was determined according to our previously described method (Yan et al., 2016). Namely, drug-treated MPC5 cells were harvested and then incubated with 50 μM DCFH-DA at 37°C for 20 min in the dark. Samples of at least 10,000 cells were measured using a FACSCalibur flow cytometer with an excitation wavelength of 488 nm and an emission of 530 nm.

### Statistical Analysis

All data are expressed as mean ± standard deviation (SD). Differences were analyzed by using SPSS 16.0 software with Turkey's test. A *p*-value of <0.05 was considered statistically significant. Immunofluorescence images and Western blotting images were analyzed by Image-Pro Plus 6.0 (Media Cybernetics, MD, USA).

## Results

### Preparation and Characterization of WED-Loaded Micelles

**Figure 2:**
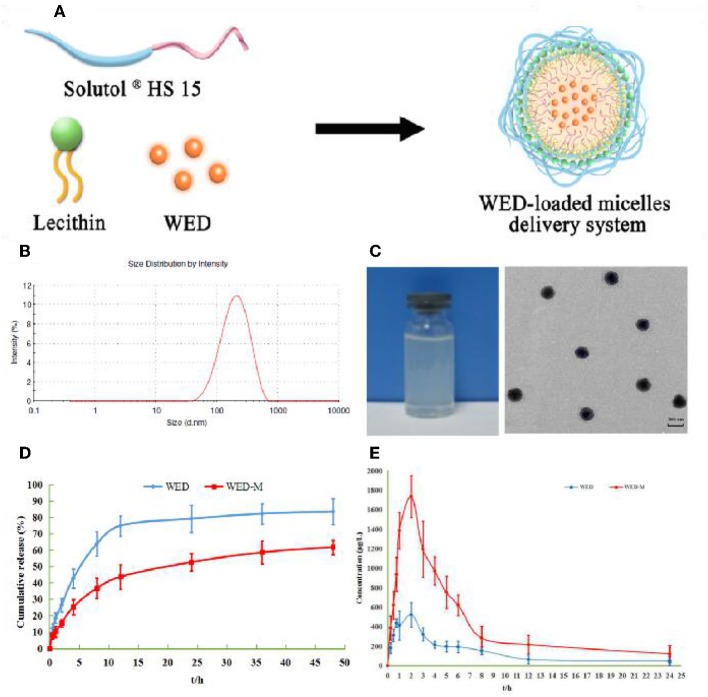
Preparation and characterization of WED-M. **(A)** WED-M was prepared using Solutol^®^ HS 15 and lecithin with the thin-film hydration method; **(B)** size distribution of WED-M; **(C)** TEM micrographs and image of WED-M, Scale bar = 200 nm, Magnification × 60,000; **(D)** *in vitro* release profiles of WED-M and free WED at 37°C, and **(E)** the plasma concentration–time curve of IS in rats after oral administration of WED and of WED-M (50 mg/kg, IS). Data are presented as mean ± SD of six individual experiments.

WED-loaded micelles were prepared with Solutol^®^ HS15 and lecithin to form a micelle system according to the schematic diagram shown in Figure 2A. Transmission electron microscopy showed the morphology of WED-M binary micelles to be spherical with uniform particle size and without aggregation (Figure 2C). As shown in Table 1 and Figure 2B, the average particle size of WED-M was 160.5 ± 3.4 nm, which basically conforms to the micelle particle size. The polydispersity coefficient (PDI) value of WED-M was 0.244 ± 0.007, indicating that the particle size was relatively uniform. A zeta potential of −30.1 ± 0.9 mV was detected by dynamic light scattering. In this study, HPLC was conducted to detect entrapment efficiency, drug loading, and solubility. An entrapment efficiency of 94.41 ± 1.64% could be achieved with drug loading of 8.6 ± 0.3%. In addition, the solubility of WED-M can reach up to 1.9 ± 0.1 mg/ml. It was obvious from these data that Solutol^®^ HS15 and lecithin can promote the formation of WED-M with good micellar characteristics.

**Table 1:** Characteristics of WED-loaded binary mixed micelle system.

**Binary mixed micelles**	**Size (nm)**	**PDI**	**Zeta potential (mV)**	**Entrapment efficiency (%)**	**Drug loading (%)**	**Solubility (mg/ml)**
WED-M	160.5 ± 3.4	0.244 ± 0.007	−30.1 ± 0.9	94.41 ± 1.64	8.58 ± 0.25	1.89 ± 0.06

*Data are presented as mean ± SD of five individual experiments*.

### WED-Loaded Micelles Have Continuous Stability

Stability is very important to the micelle system. As shown in Table 2, the stability of the WED-M formed was examined within 14 days based on the determination of solubility, average size, PDI, zeta potential, and clarity. All of the detection indicators were stabilized within 14 days. These results indicated that the prepared WED-M remained a stable system during this period of determination.

**Table 2:** Stability of WED-loaded micelles.

**Time (days)**	**Solubility (mg/ml)**	**Average size (nm)**	**PDI**	**Zeta potential (mV)**	**Clarity**
1	1.88 ± 0.04	157.3 ± 2.3	0.237 ± 0.007	−30.5 ± 0.5	+++
3	1.89 ± 0.05	159.2 ± 1.7	0.244 ± 0.008	−29.7 ± 0.6	+++
7	1.85 ± 0.09	161.4 ± 2.2	0.251 ± 0.005	−30.1 ± 0.9	+++
14	1.84 ± 0.07	164.1 ± 2.6	0.246 ± 0.008	−29.9 ± 0.7	+++

*Data are presented as mean ± SD of five individual experiments*.

### Micelles Sustainedly Release WED Molecules

Micelles may affect the release behavior of WED molecules. As depicted in Figure 2D, WED-M and WED exhibit different release properties in dialysis under sink conditions. Free WED molecules were released rapidly to 74.8 ± 6.2% within 12 h, whereas the release of WED-M only reached 43.71 ± 7.53%. After 48 h of full release, the amount released from WED-M reached 61.88 ± 4.40%, which was lower than the 83.56 ± 7.92% of free WED molecules. It was obvious that the release curve of WED-M was gentler and slower than that of free WED molecules. The data showed that micelles formed by Solutol^®^ HS15 and lecithin might affect the release of WED, having the property of sustained release.

### Micelles Promote the Permeability of WED in Caco-2 Cells

A Caco-2 cell model was used to evaluate the permeability and efflux ratio of WED and WED-M. As listed in Table 3, the Papp AP to BL value of WED-M was (36.6 ± 1.8) × 10^−6^ cm/s, while that of WED was (20.21 ± 1.01) × 10^−^6 cm/s, indicating a significant 1.61-fold higher Papp AP to BL ratio than WED only. However, the Papp BL to AP value of WED-M was only 72% of that of WED. After being prepared into micelles, the efflux ratio of WED was decreased significantly from 1.37 to 0.61, only 44.52% of that of free WED molecules. This result suggested the WED-M could enhance the permeability of WED in Caco-2 cells.

**Table 3:** Permeability and efflux ratio of WED and WED-loaded micelles.

**Compound/micelles**	**P_app_*AP* → *BL*/10^−6^ cm/s**	**P_app_*BL* → *AP*/10^−6^ cm/s**	**Efflux ratio**
WED	20.21 ± 1.01	27.68 ± 1.43	1.37
WED-loaded micelles	26.59 ± 1.77^*^	24.91 ± 1.83^#^	0.94^Δ^

*Data are presented as mean ± SD of three individual experiments. Absorption permeability is expressed as P_app_AP → BL, whereas secretory permeability is presented as P_app_BL → AP. Efflux radio is P_app_ (P_app_BL → AP)/Papp (P_app_AP → BL)*.

* *P < 0.05 compared with the P_app_AP → BL of WED*.

# *P < 0.05 compared with the P_app_BL → AP of WED*.

Δ *P < 0.05 compared with the efflux ratio of WED*.

### WED-M Enhances the Bioavailability of WED

The pharmacokinetic parameters and mean plasma concentration-time profiles are presented in Table 4 and Figure 2E. Herein, we noticed that the average value of AUC_0−t_ (AUC: area under the curve) of WED-M was 8122.9 ± 567.5 μg/L/h, while that of WED was 2922.3 ± 252.7 μg/L/h, demonstrating that the bioavailability of WED was increased 2.78-fold. The average value of C_max_ was enhanced from 519.37 ± 53.59 μg/L of WED to 1461.03 ± 124.46 μg/L of WED-M, an increase of 281.5%. The highest peak time (T_max_) of WED-M was similar to that of WED alone. In a similar manner to C_max_, the average value of t_1/2_ was extended from the 3.02 ± 1.79 h of WED to 4.69 ± 1.87 h for WED-M, an increase of 155.5%. These results regarding the pharmacokinetic characteristics of WED and WED-M indicated that the micelles markedly improved the bioavailability of WED, with a sustained release.

**Table 4:** Pharmacokinetic parameters of WED and WED-M (50 mg/kg).

**Parameter**	**WED**	**WED-M**
AUC_0−t_ (μg/L/h)	2922.36 ± 252.74	8122.88 ± 567.46^**^
AUC_0−∞_ (μg/L/h)	3439.99 ± 290.21	9886.54 ± 549.00^**^
MRT_0−t_ (h)	7.87 ± 0.28	7.40 ± 0.55
MRT_0−∞_ (h)	12.08 ± 1.28	12.75 ± 0.73
t_1/2_ (h)	3.02 ± 1.79	4.69 ± 1.87
T_max_ (h)	1.93 ± 0.48	1.98 ± 0.25
C_max_ (μg/L)	519.37 ± 53.59	1461.03 ± 124.46^**^

*Data were obtained from six individuals and are presented as mean ± SD*.

** *P < 0.01 compared with WED*.

### Improvement of WED-M on DOX-Induced Kidney Damage

**Figure 3:**
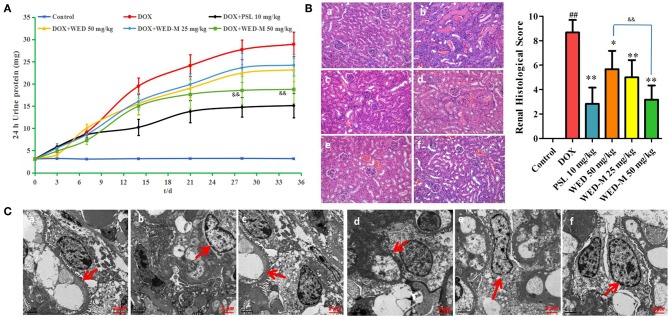
Effect of WED-M on urinary protein excretion at each time point **(A)**. Mice were treated orally with 50 mg/kg of WED, WED-M at doses of 25 and 50 mg/kg, or 10 mg/kg of prednisone acetate (PSL) after injection of doxorubicin (DOX, 7.5 mg/kg). Urine was collected for determination of proteinuria on days 3, 7, 14, 21, 28, and 35 after DOX injection. **(B)** Effects of WED-M on kidney pathology in DOX-induced nephropathy mice. Histopathological image of kidney tissue was evaluated for pathological scores. Magnification × 200. **(C)** Ultrastructure observation of kidney by transmission electron microscope. Magnification × 10,000. (a) control; (b) DOX; (c) DOX plus PSL; (d) WED at 50 mg/kg plus DOX; (e) WED-M at 25 mg/kg plus DOX; (f) WED-M at 50 mg/kg plus DOX. Data were obtained from six individuals and are presented as mean ± SD. ^##^*P* < 0.01, compared with Control. ^*^P < 0.05, ^**^P < 0.01, compared with DOX; ^&&^*P* < 0.01, compared with WED.

The efficacy of WED and WED-M was investigated to show the improvement of micelles on WED as regards DOX-induced kidney damage and renal failure. As shown in Figure 3A, exposure to 7.5 mg/kg DOX increased the 24-h urinary protein exception level remarkably when compared with normal control (*P* < 0.01). This increase was inhibited significantly by treatment with WED or WED-M (*P* < 0.05; *P* < 0.01). Interestingly, WED-M has a more significant down-regulation on 24-h urinary protein exception than WED (*P* < 0.05; *P* < 0.01).

Multiple biochemical parameters for kidney function, including TG, TC, ALB, TP, Scr, and BUN, were significantly altered after administration of DOX. As shown in Table 5, compared with DOX, both WED and WED-M could decrease the levels of TG, TC, Scr, and BUN significantly, whereas ALB and TP increased markedly (*P* < 0.05; *P* < 0.01). By comparing the results of WED and WED-M, it can be found that WED-M is superior to WED in regulating all of the biochemical indexes, with significant difference (*P* < 0.05; *P* < 0.01).

**Table 5:** Comparison of the biochemical parameters of WED-M and WED in DOX-induced renal damage.

**Group**	**TG**	**TC**	**ALB**	**TP**	**Scr**	**BUN**
	**(mmol/L)**	**(mmol/L)**	**(g/L)**	**(g/L)**	**(μmol/L)**	**(μmol/L)**
Control	0.97 ± 0.13	1.39 ± 0.22	32.71 ± 1.93	62.24 ± 3.97	50.04 ± 2.57	7.01 ± 0.56
DOX	4.54 ± 0.81^##^	4.92 ± 0.94^##^	17.01 ± 2.04^##^	31.91 ± 4.11^##^	78.97 ± 8.33^#^	14.98 ± 1.91^##^
PSL 10 mg/kg	2.77 ± 0.68^**^	2.53 ± 0.47^**^	26.98 ± 2.11^**^	50.43 ± 4.54^**^	59.22 ± 7.44^*^	9.97 ± 1.47^*^
WED 25 mg/kg	3.37 ± 0.42^*^	3.85 ± 0.48^*^	21.04 ± 2.59^*^	42.58 ± 4.62^*^	69.17 ± 6.29^*^	11.45 ± 1.31^*^
WED 50 mg/kg	2.83 ± 0.45^*^	3.23 ± 0.62^*^	22.99 ± 3.03^*^	47.66 ± 5.97^*^	65.43 ± 7.67^*^	10.61 ± 1.24^*^
WED-M 50 mg/kg	1.86 ± 0.39^**^^ΔΔ^	2.57 ± 0.53^**^^Δ^	27.54 ± 3.88^*^^Δ^	54.27 ± 6.57^*^^ΔΔ^	57.93 ± 8.21^*^^Δ^	8.08 ± 0.73^**^^Δ^

*Data were obtained from six individuals and are presented as mean ± SD*.

# P < 0.05 and

## P < 0.01 compared with Control;

* P < 0.05 and

** P < 0.01 compared with DOX;

Δ P < 0.05 and

ΔΔ *P < 0.01 compared with WED*.

Pathological changes in kidney tissue were detected by HE staining (Figure 3B), and the ultrastructure was examined by transmission electron microscope (Figure 3C). We observed obvious pathological damage to renal tissue in DOX-induced rats, with glomerular focal necrosis, damaged glomerular structure, hyperplasia of renal tubular epithelial cells, dilatation of renal tubular cells, and infiltration of inflammatory cells to renal interstitial cells. Results showed a significant improvement with WED-M compared to free WED molecules on pathological damage.

### WED-M Inhibits ROS Elevation in DOX-Stimulated Podocytes

**Figure 4:**
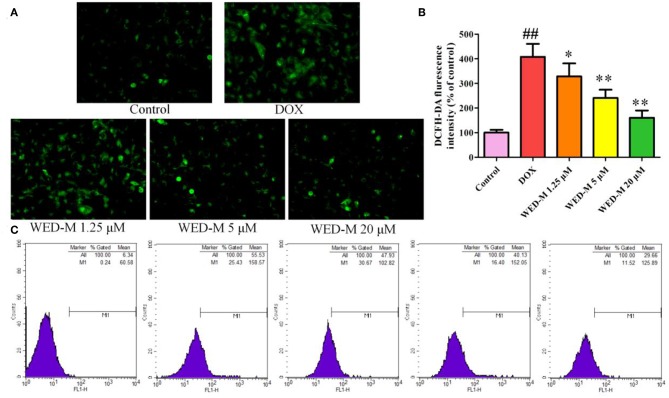
Effect of WED-M on intracellular ROS production in DOX-induced podocytes. **(A)** DCFH-DA fluorescence was detected by fluorescence microscope. **(B)** Integrated optical density of DFCH-DA images. Integrated optical density was evaluated with Image-Pro Plus 6.0. **(C)** Reactive oxygen species were measured by flow cytometry. Data are presented as mean ± SD of six individual experiments. ^##^*P* < 0.01, compared with Control. ^*^P < 0.05, ^**^P < 0.01, compared with DOX.

Both DCFH-DA fluorescence and flow cytometry were conducted to observe the level of ROS in MPC5 podocytes. As depicted in Figures 4A,B, the level of intracellular ROS was promoted obviously by exposure to 4 μM DOX compared with blank control (*P* < 0.01). Interestingly, the rising level of ROS was weakened meaningfully by treatment with WED-M at concentrations of 1.25, 5, and 20 μM in a dose-dependent manner (*P* < 0.05; *P* < 0.01). The results of flow cytometry also showed a similar trend in the reduction of WED-M to the generation of ROS (Figure 4C). Both results were indicative of the regulation effect of WED-M on oxidative stress damage.

## Discussion

**Figure 5:**
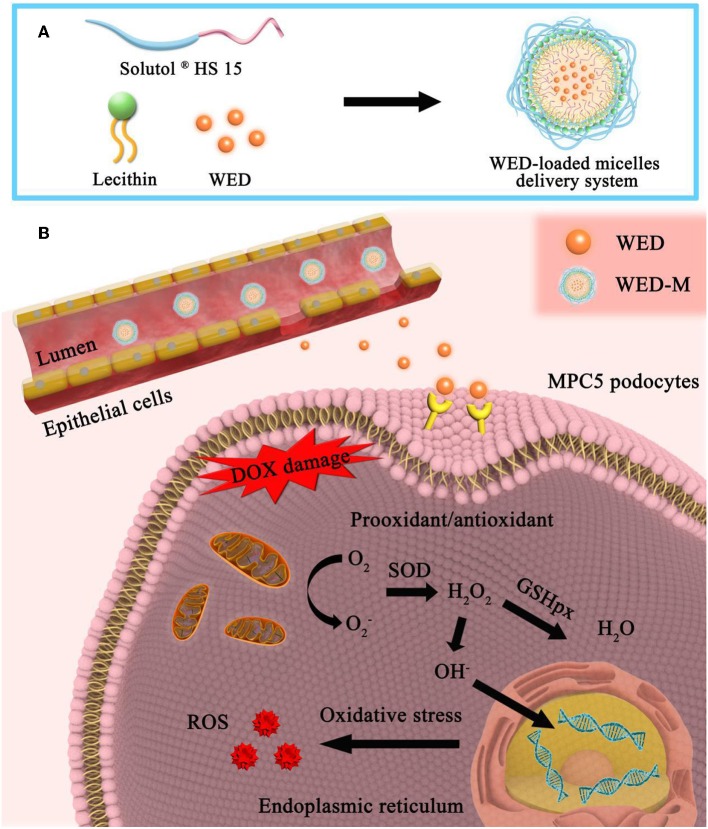
Formation, permeability, and sustainable release of WED-M and its major signaling pathways. **(A)** Formation of MED-loaded mixed micelles. A WED-loaded micelle drug delivery system was formed by the combination of Solutol^®^ HS 15 and Lecithin at a ratio of 7:3. **(B)** Mechanism diagram of WED-M. In the intestine, these excipients can enhance the release of WED molecules from the micelles with sustained release so as to penetrate the intestinal epithelial cells and can also enhance the permeability of WED to the cell membrane of targeting cells. DOX-induced prooxidant/antioxidant imbalance was regulated by the enhanced permeability and bioavailability of WED-M molecules. The DOX-induced intracellular oxidative stress response was attenuated by WED-M, eliminating stress-produced free radicals. WED-M can further elicit intrinsic reactive oxygen species (ROS)-generation, which provides the host with protection against DOX exposure provoking and triggering stress injury in podocyte MPC5 cells.

It has been shown that the poor permeability and bioavailability of insoluble drugs affect their absorption in the body, thus affecting their efficacy. The low bioavailability of drugs due to poor absorption and water-insoluble properties limits their application in clinical contexts. Research suggests that about 40% of the marketed drugs and 90% of drug candidates exhibited poor absorption in the gastrointestinal tract due to poor water solubility, resulting in limited or poor efficacy (Shen et al., 2019). The *in vivo* behavior and bioavailability of these compounds could be influenced by both physicochemical and physiological factors (Upadhyay et al., 2012). WED, a naturally occurring coumestan, has poor absorption through the intestine (Chatzidaki et al., 2017). In the present study, the drug delivery system of WED-M was developed by encapsulating Solutol^®^ HS15 and lecithin, which could improve the water solubility and bioavailability of WED. The improved pharmacokinetics and pharmacodynamics of WED-M vs. free WED molecules were characterized, and the underlying mechanism of WED-M was explored to reveal the enhanced bioavailability and signaling pathway of the WED complex (Figure 5).

In our previous study, the formulation of the micelle delivery system was established based on screening adjuvants (Trimaille et al., 2019). In the pre-experiment, the effects of different proportions of excipients on micellar particle size and PDI were compared to optimize the prescription. We observed a three-peak distribution of the particle size of mixed micelles when the ratio of Solutol^®^ HS15 to lecithin was 9:1 and 8:2, respectively, indicating the inhomogeneity of micelles because of the poor integration of two adjuvants. After the ratio was changed to 6:4 or 5:5, the particle size of the micelles was more than 300 nm, and the dispersion coefficient was more than 0.4, with a non-uniform distribution. The proportion of 7:3 formed the ideal mixed micelles, with a uniform mean particle size of about 160 nm and a dispersion coefficient of 0.244, demonstrating a combination of Solutol^®^ HS15 and lecithin that is an improved prescription for micellar preparation. Examination with transmission electron microscopy showed the WED-Mto have a spherical morphology without aggregation. Our data suggested that a 7:3 ratio is a reasonable combination of Solutol^®^ HS15 and lecithin to form a WED-loaded micelle delivery system.

Solutol^®^ HS 15 was employed to enhance the stability of WED-M. A small proportion of the 12-hydroxyl group in the chemical structure of Solutol^®^ HS 15 can be etherified by polyethylene glycol. Under 25°C conditions, the stability of Solutol^®^ HS 15 can be maintained for 24 months (Shen et al., 2019). It is noteworthy that Solutol^®^ HS 15 does not show significant property alteration, even with hydrolysis at 121°C for 15 min. We examined the stability of WED-M over 14 days. The values of solubility, average size, PDI, and zeta potential showed sustained WED-M stability. The higher absolute value of zeta potential indicated that the greater amount of charge of the colloid could be the cause of the greater stability of the colloidal dispersion system.

In order to evaluate delivery enhancement, the permeability, release behavior, and pharmacokinetic characteristics of WED-M and free WED molecules were compared. The results showed that the mixed micelles could significantly increase the permeability of WED molecules and inhibit the efflux ratio in Caco-2 cells compared with free WED molecules. The release profile showed that the micelle delivery system was capable of sustained release of WED molecules and of achieving an improved bioavailability. The sustained-release characteristics may be attributed to the interaction of water molecules and lipophilic groups of the surfactant with the repulsive force that is greater than the attractive force, forming the aggregate of surfactant molecules via van der Waals forces to achieve sustained-release WED molecules. Under *in vitro* dialysis, the highest release behavior of WED-M is less than that of WED. However, the Cmax of WED-M was enhanced to 2.78 times that of WED due to the improved permeability of the micelle delivery system.

Oxidative damage is considered to be an important pathogenic factor in DOX-induced nephrotoxicity. The exposure to DOX can induce oxidative stress in renal tissues, accompanied by a significant change of multiple oxidation indicators (Liu et al., 2018). Interestingly, not only does WED have antioxidant activity, but also the micellar delivery system can act as an antioxidant carrier. The delivery system of micelles is composed of a mixture of surfactants, lecithin, and natural antioxidants (Li et al., 2017). Increasing evidence shows that the extraction of lecithin from egg yolk has potential health-enhancing antioxidant properties involving superoxide anion and hydroxyl radical scavenging and ferrous chelation (Dalmazzo et al., 2019). Another study demonstrated that the antioxidant activity of lecithin emulsifier significantly influenced the permeation of free radicals across the emulsion interface (Chung et al., 2019). In the drug delivery system of WED-M, these adjuvants act as an antioxidant carrier, potentially synergistic with the antioxidant effects of WED.

The natural compound WED was isolated from the medical herb Eclipta alba and has been shown to attenuate chemotherapy agent-induced renal dysfunction and tubular injury from nephrotoxicity (Kobori et al., 2004; Miao et al., 2018). Importantly, recent evidence has demonstrated that the induction of WED on the abnormal proliferation of human renal mesangial cells might be associated with regulating the activity of several key members of the NF-κB signaling pathway (Ali et al., 2016). In this study, the drug delivery system of WED-M showed better regulation of renal function, including TG, TC, Scr, BUN, ALB, and TP than its free molecules. All the results, including the 24-h urine protein excretion level, HE staining, and TEM observations, showed a significant improvement with WED-M over WED. These findings indicated that the improved efficacy was closely related to the increase in the permeability and bioavailability of the WED-loaded micelle delivery system.

A delivery system of WED-M was designed to improve the permeability and bioavailability of water-insoluble WED by encapsulating Solutol^®^ HS15 and lecithin. It was shown to have continuous stability and sustained release profiles. This study provided new insight into the effect of WED-M on the treatment of chemotherapy agent-induced renal injury.

## Data Availability Statement

All datasets generated for this study are included in the article/supplementary material.

## Ethics Statement

The animal study was reviewed and approved by Ethics Committee of China Pharmaceutical University; Affiliation: China Pharmaceutical University.

## Author Contributions

LF and ZL designed the experiment and analyzed the data. LW and XL wrote the manuscript. YC and BY conducted the experiment. DY and YL contributed to the resources access. XH, JL and SD contributed to data interpretation and commented on the manuscript. XJ conceived and supervised the overall project. All authors listed have made a substantial, direct and intellectual contribution to the work, and approved it for publication.

### Conflict of Interest

The authors declare that the research was conducted in the absence of any commercial or financial relationships that could be construed as a potential conflict of interest.
